# Supplementary material for: Recombination between heterologous human acrocentric chromosomes
Source: Nature. 2023 May 10;617(7960):335–43. doi: 10.1038/s41586-023-05976-y (PMC10172130; doi:10.1038/s41586-023-05976-y)
Supplement: Supplementary file 1 — This file contains Supplementary Figs. 1–29. [file 41586_2023_5976_MOESM1_ESM.pdf]

nature portfolio

https://doi.org/10.1038/s41586-023-05976-y

Supplementary information

Recombination between heterologous human acrocentric chromosomes

In the format provided by the authors and unedited

Supplementary Figures for
Recombination between heterologous human acrocentric chromosomes

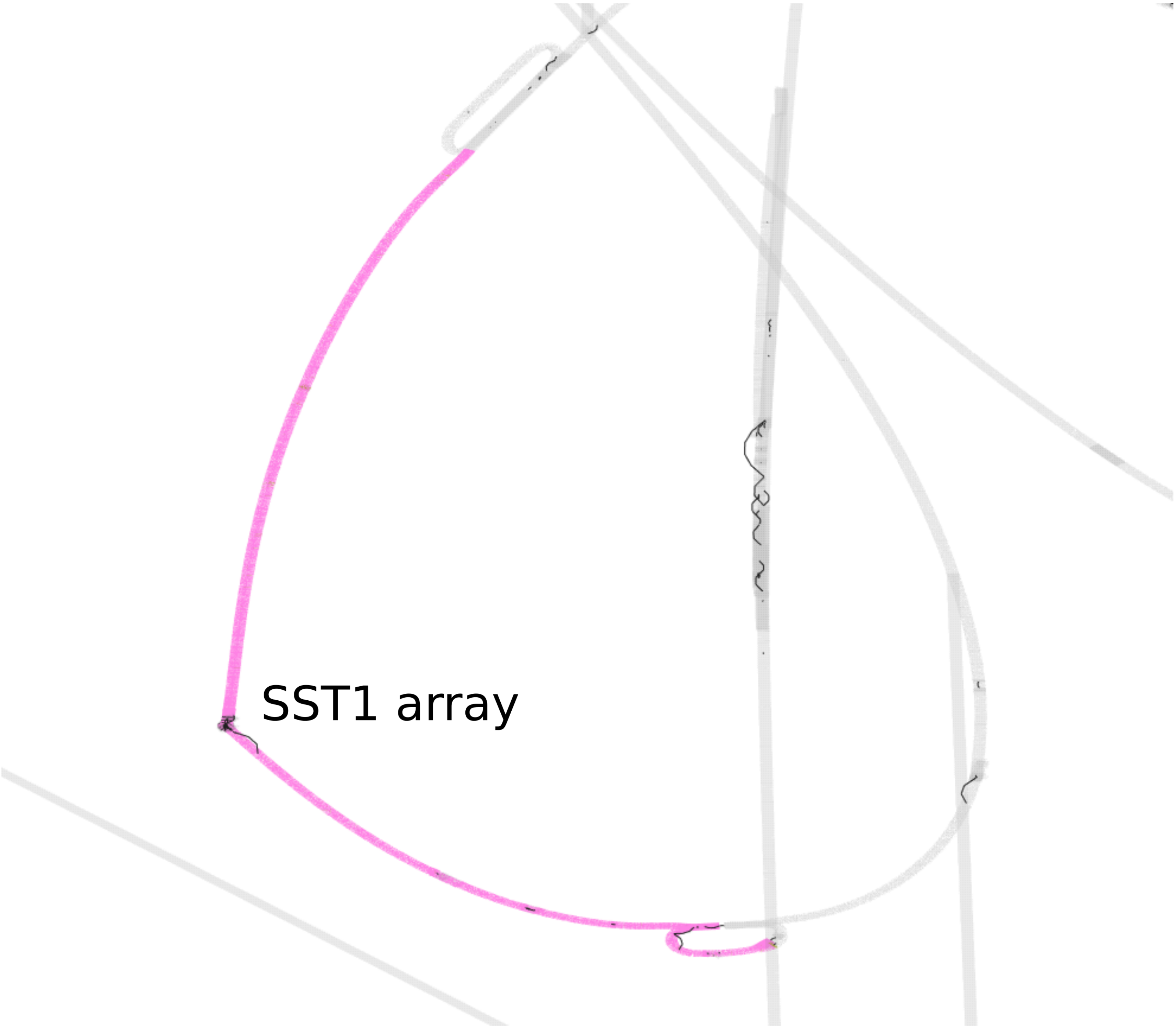

Supplementary Figure 1: Regions of a PVG built from centromere-spanning HPRCy1 contigs plus T2T-CHM13 and GRCh38 references. We apply GFAESTUS to visualize the 2D layout generated by ODGI in the PGGB pipeline. We focus on the segmentally duplicated core centered in the SST1 array (labeled).

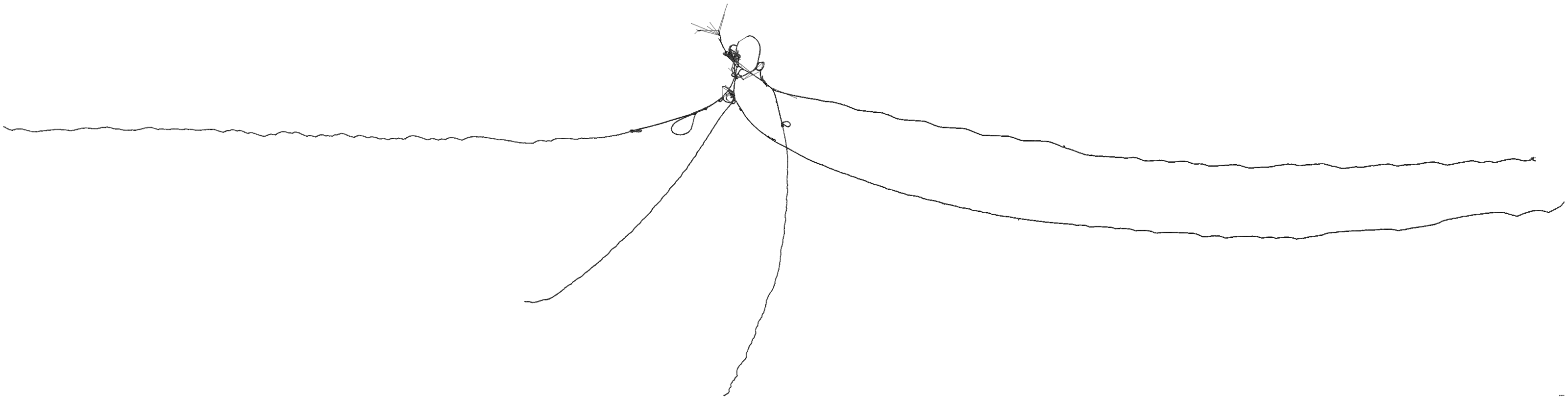

Supplementary Figure 2: A PVG built from centromere-spanning HPRCy1 contigs, without embedding the T2T-CHM13 and GRCh38 references. We apply ODGI to visualize the 2D layout generated in the PGGB pipeline. This renders sequences and chains of small variants as linear structures, while repeats caused by segmental duplications, inversions, and other structural variants tend to form loops and tangles. The acrocentric q-arms are almost completely separated, while the p-arms unite in a structure adjacent to the rDNA array.

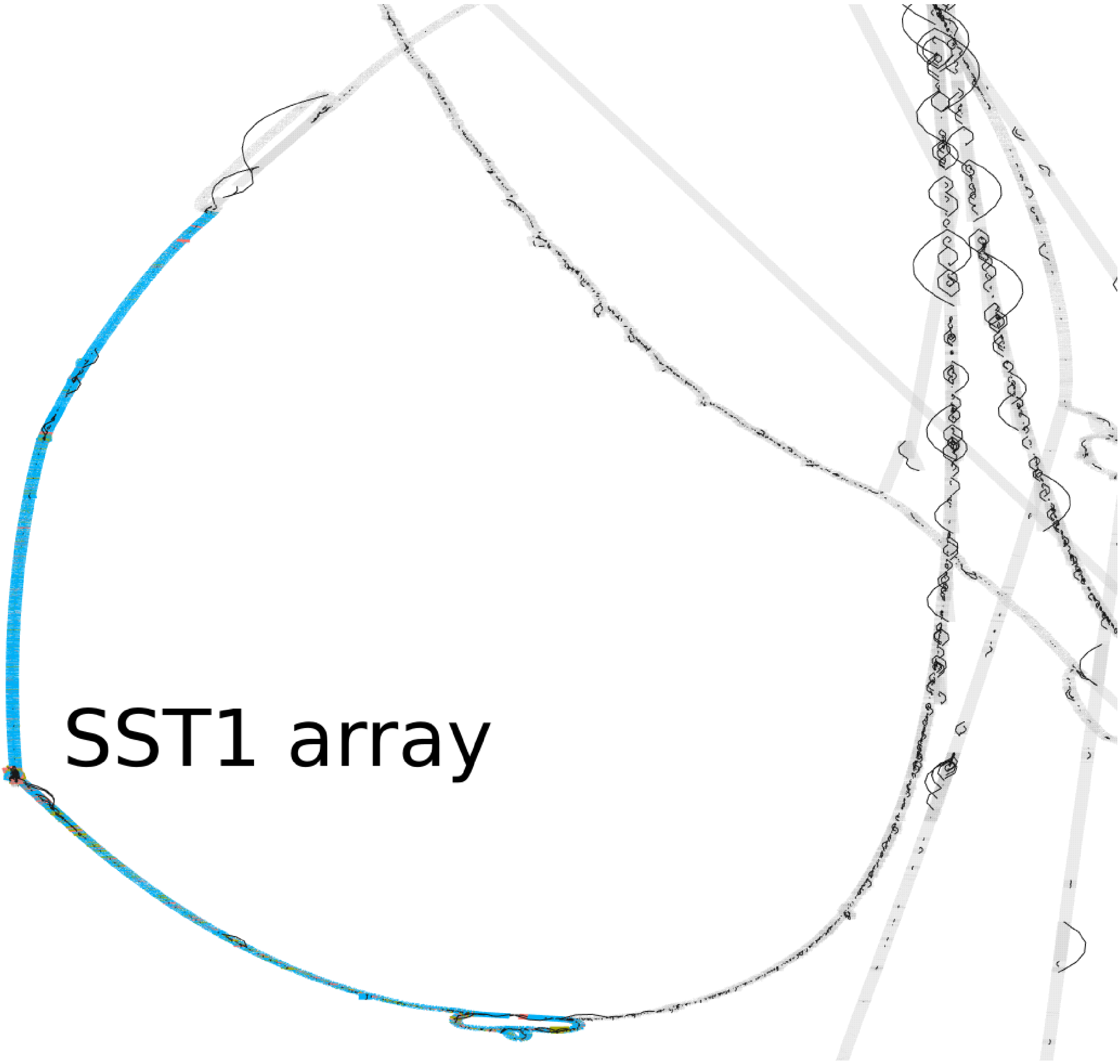

Supplementary Figure 3: Regions of a PVG built from centromere-spanning HPRCy1 contigs without the T2T-CHM13 and GRCh38 references. We apply GFAESTUS to visualize the 2D layout generated by ODGI in the PGGB pipeline. We focus on the segmentally duplicated core centered in the SST1 array (labeled).

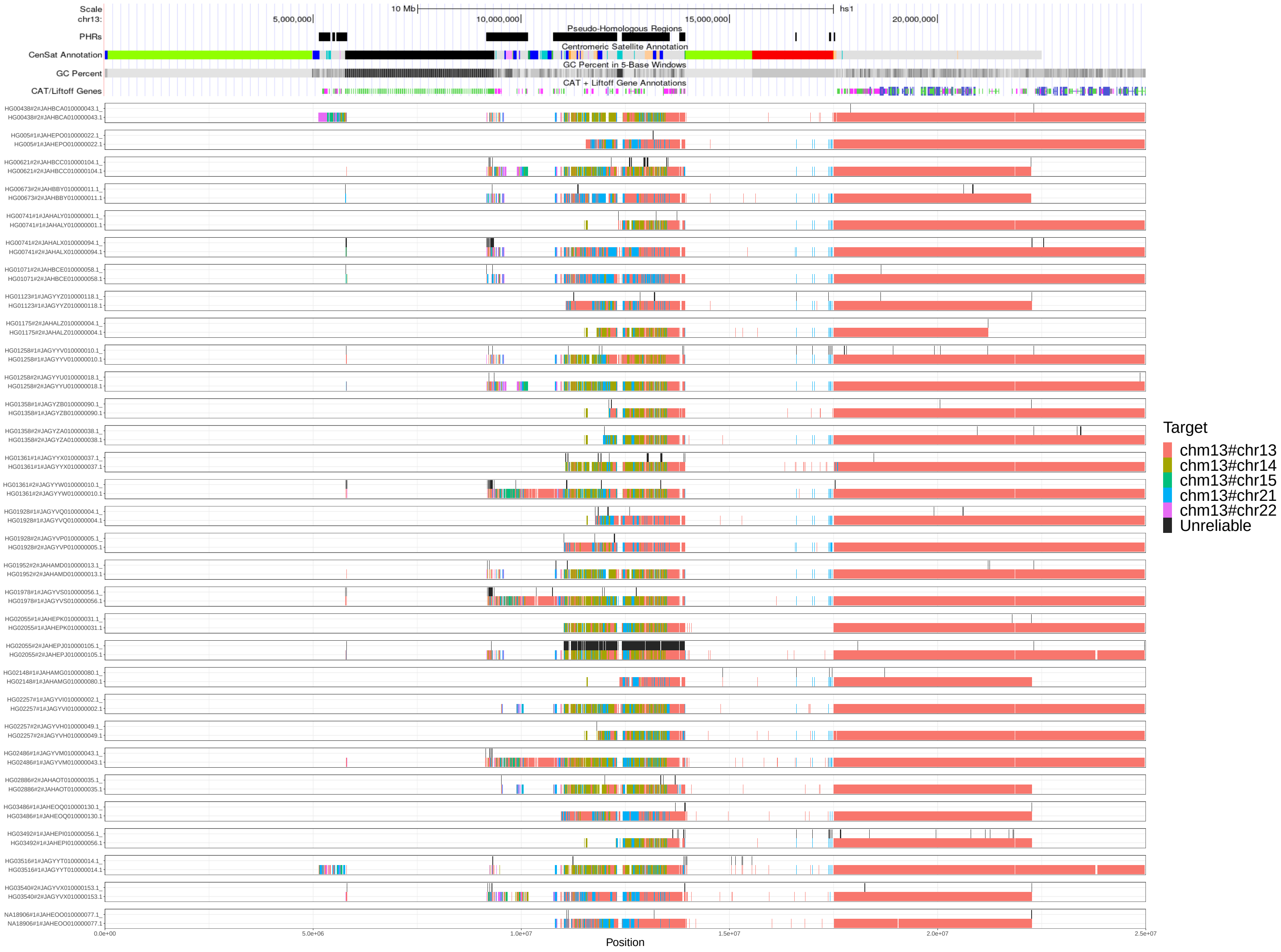

Supplementary Figure 4: Untangling of HPRCy1-acro's sequences belonging to chromosome 13 versus T2T-CHM13. We display all mappings above 90% estimated pairwise identity, without removing those covering regions classified as unreliable. Above each contig, we display the unreliable regions in black.

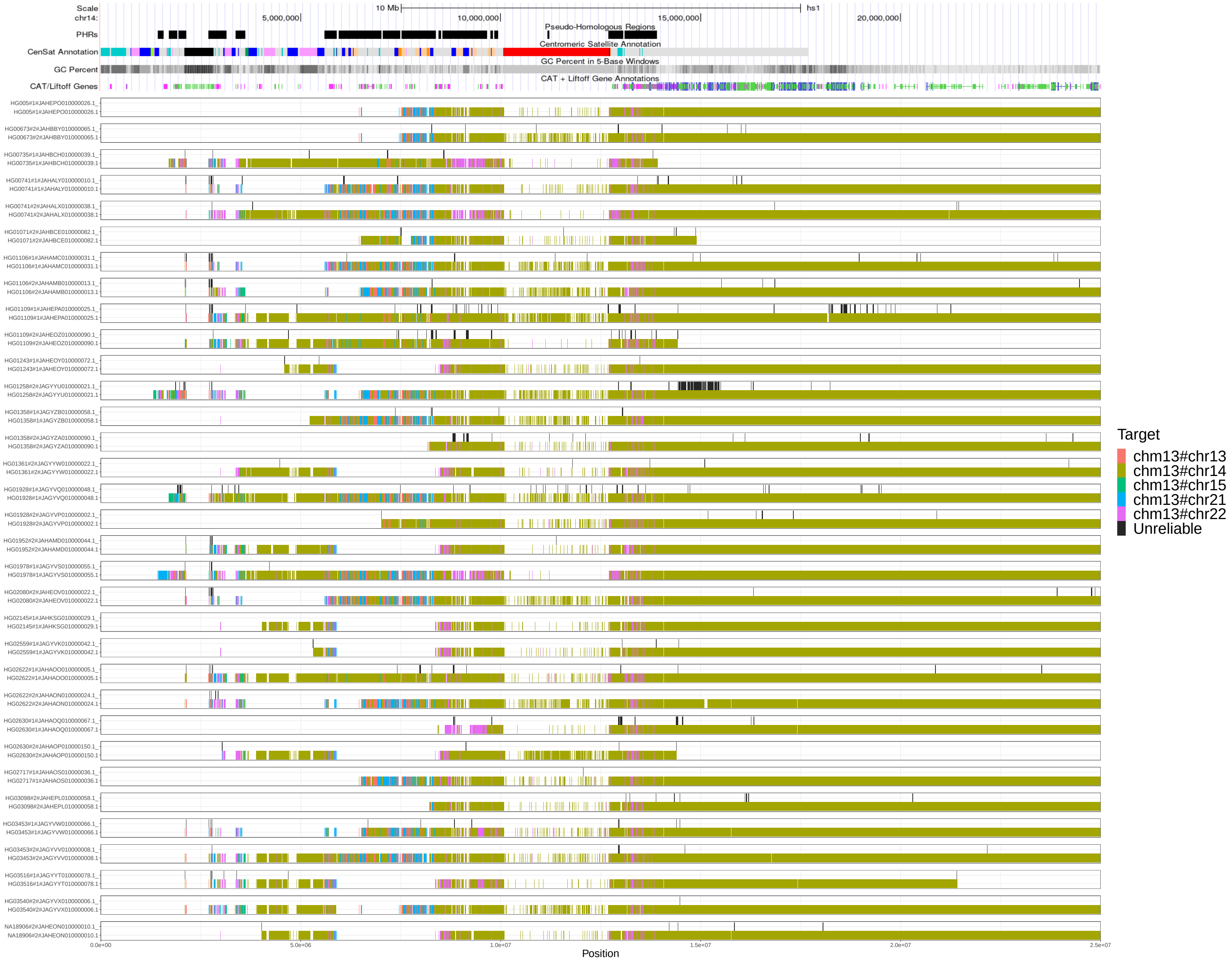

Supplementary Figure 5: Untangling of HPRCy1-acro's sequences belonging to chromosome 14 versus T2T-CHM13. We display all mappings above 90% estimated pairwise identity, without removing those covering regions classified as unreliable. Above each contig, we display the unreliable regions in black.

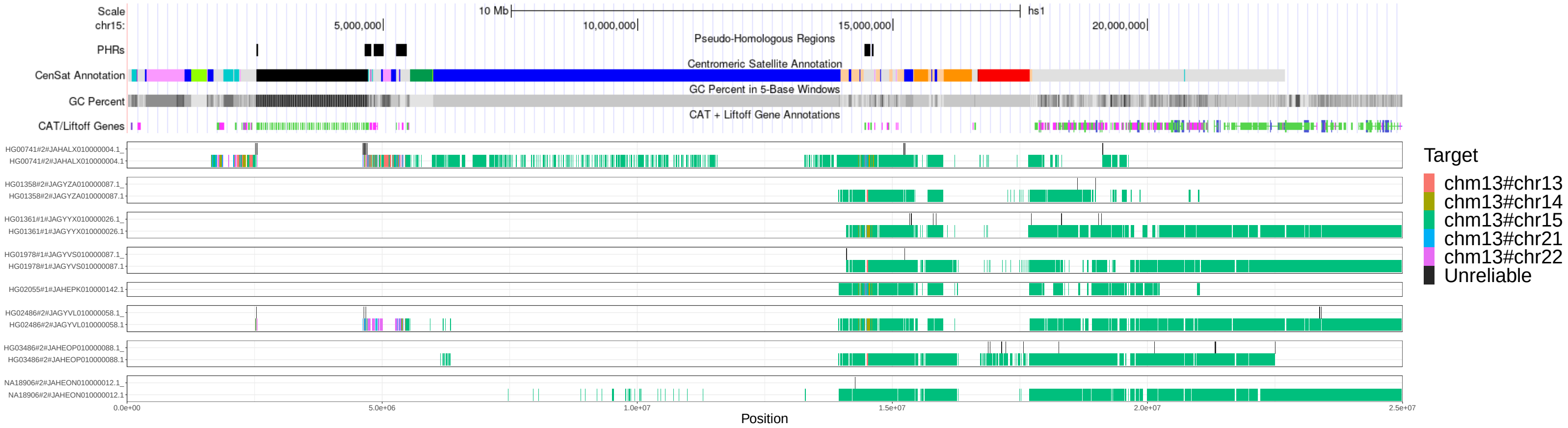

Supplementary Figure 6: Untangling of HPRCy1-acro's sequences belonging to chromosome 15 versus T2T-CHM13. We display all mappings above 90% estimated pairwise identity, without removing those covering regions classified as unreliable. Above each contig, we display the unreliable regions in black.

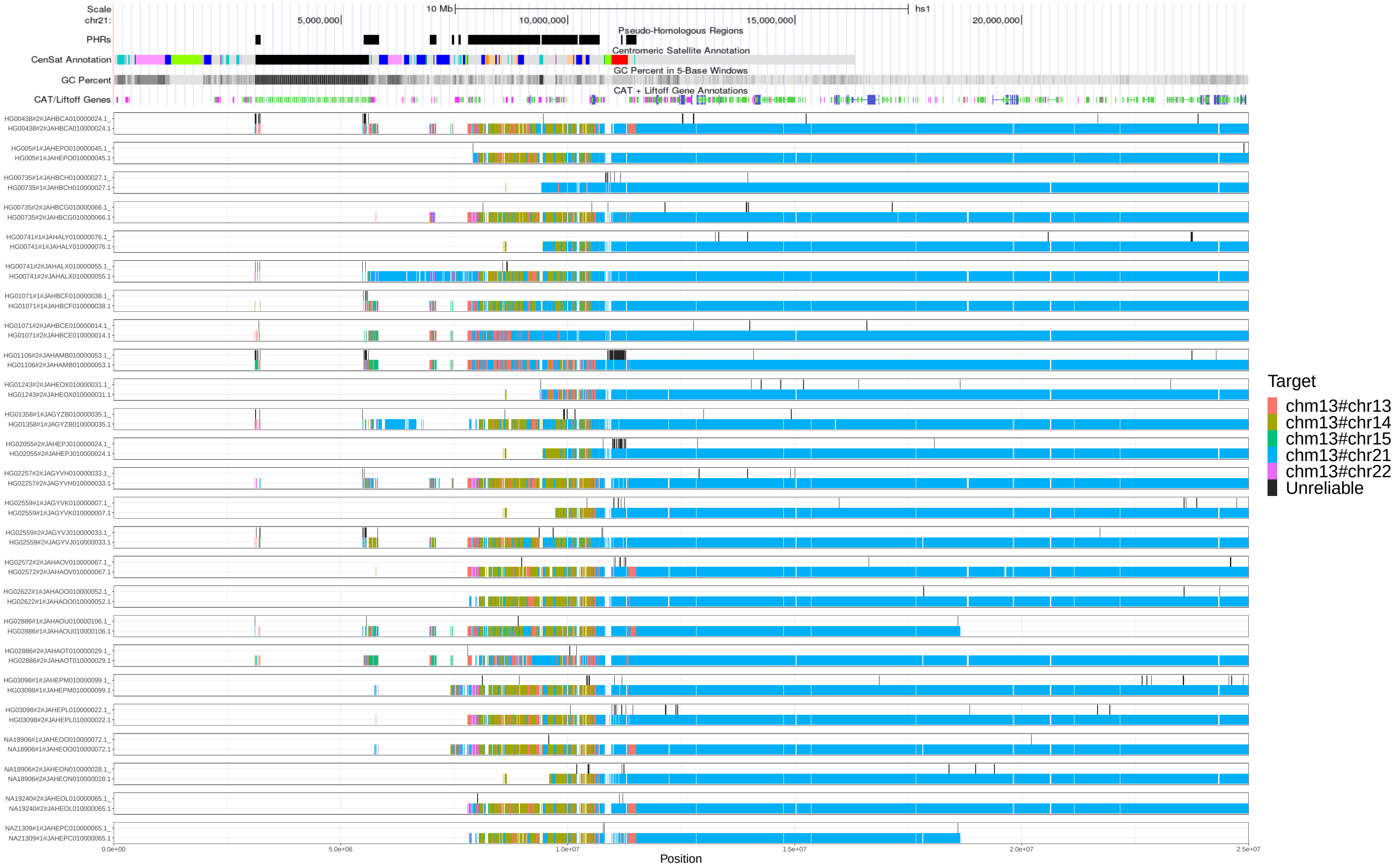

Supplementary Figure 7: Untangling of HPRCy1-acro's sequences belonging to chromosome 21 versus T2T-CHM13. We display all mappings above 90% estimated pairwise identity, without removing those covering regions classified as unreliable. Above each contig, we display the unreliable regions in black.

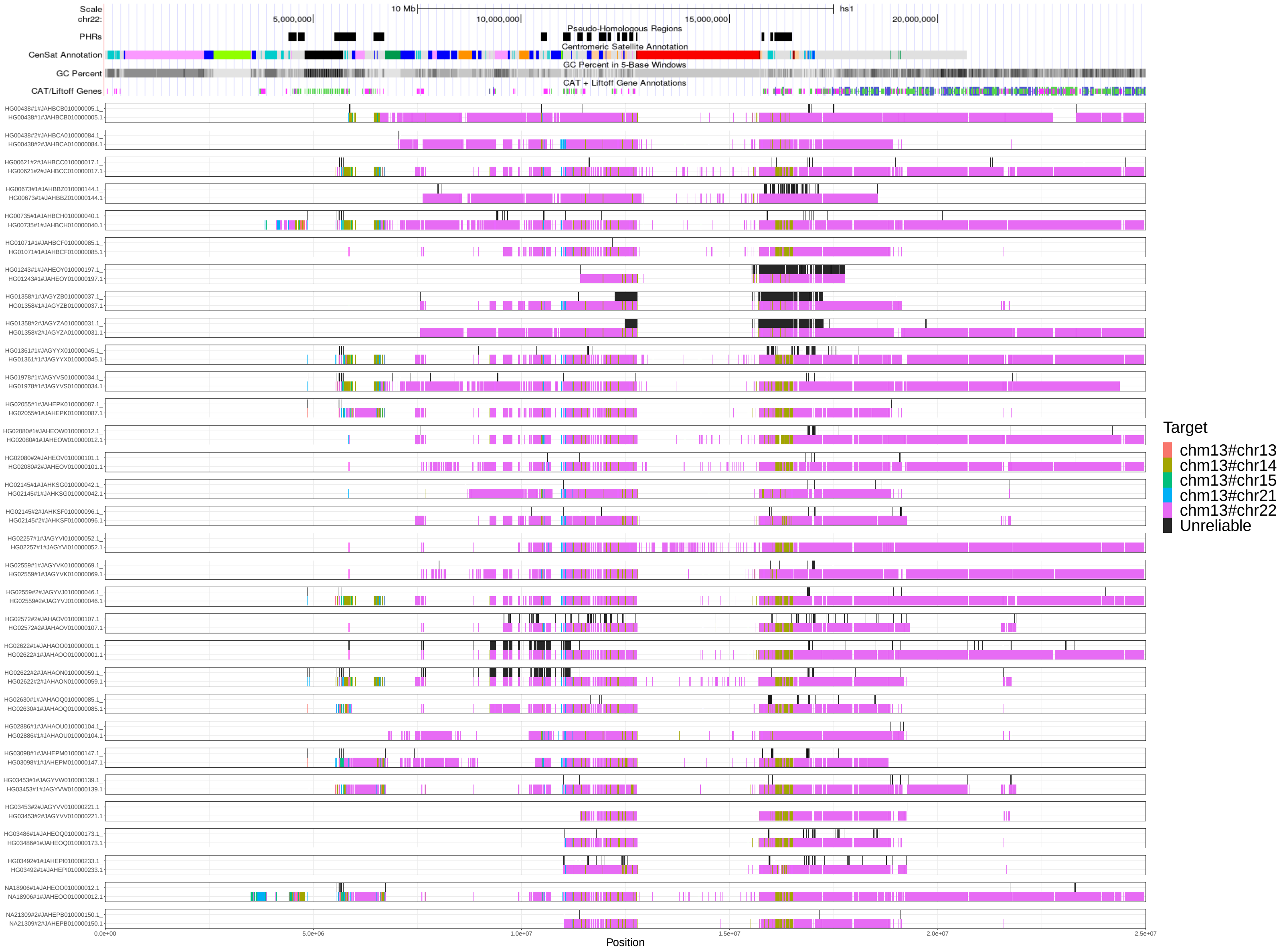

Supplementary Figure 8: Untangling of HPRCy1-acro's sequences belonging to chromosome 22 versus T2T-CHM13. We display all mappings above 90% estimated pairwise identity, without removing those covering regions classified as unreliable. Above each contig, we display the unreliable regions in black.

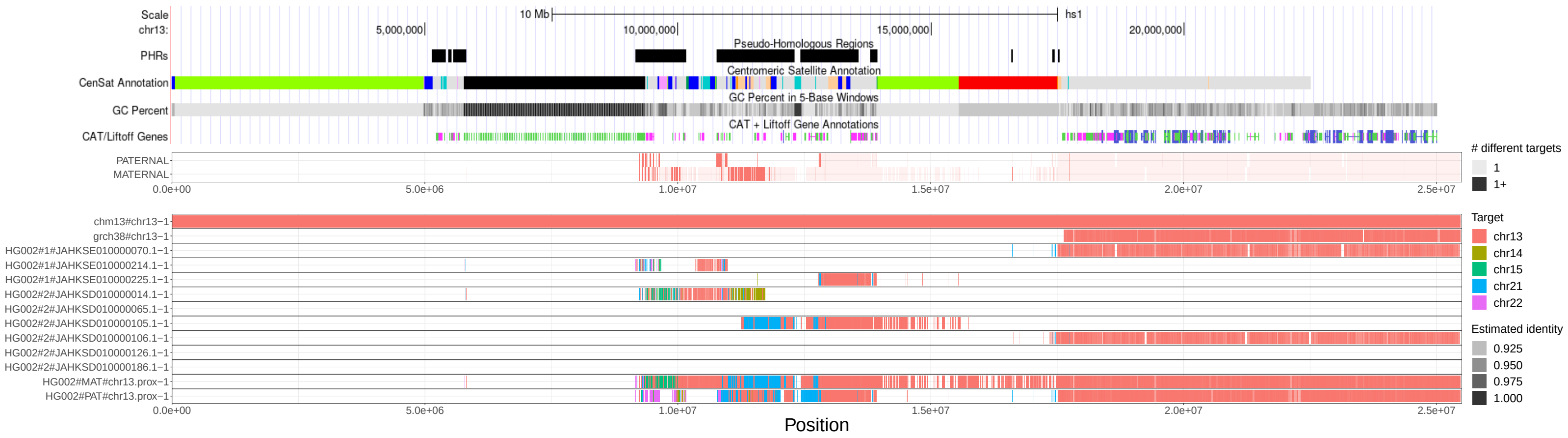

Supplementary Figure 9: For each base position of T2T-CHM13 chromosome 13, we compared the untangling best-hit of the HG002-HPRCy1 contigs with the best-hit supported by HG002-Verkko's contigs. We considered only best-hits with an estimated identity of at least 90% that cover regions labeled as reliable. We reported the number of different targets for each reference position. On the bottom, the untangling of HG002's contigs from HG002-Verkko assembly as those seen in HG002-HPRCy1 assemblies, for chromosome 13 versus T2T-CHM13. Transparency shows the estimated identity of the mappings. We display all mappings above 90% estimated pairwise identity. Checkerboard patterns observed in several regions of the SAACs correspond to contexts that may permit ongoing recombination.

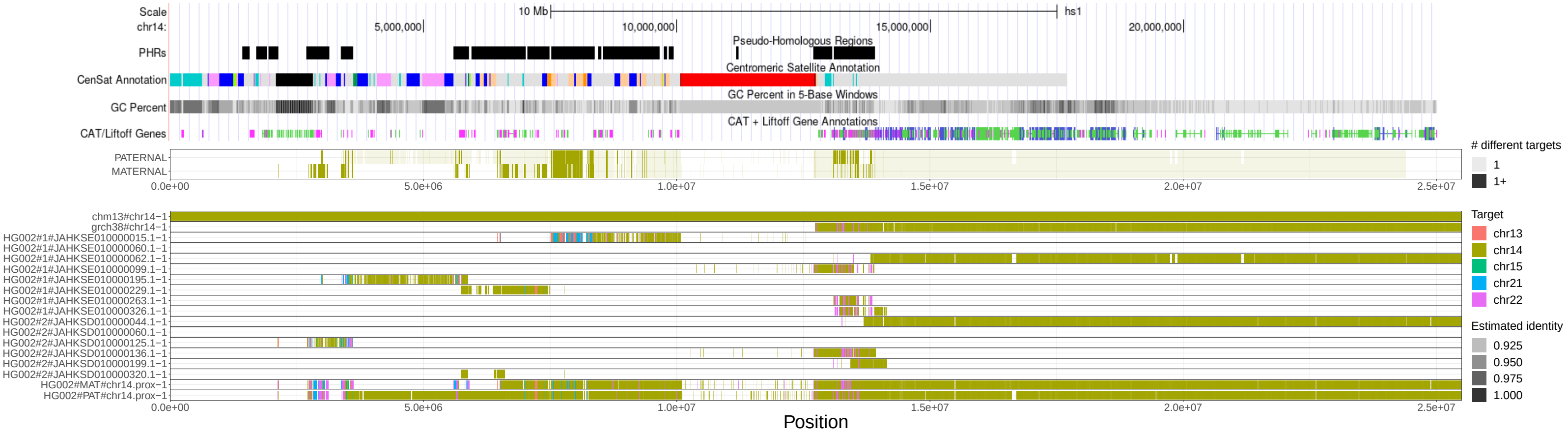

Supplementary Figure 10: For each base position of T2T-CHM13 chromosome 14, we compared the untangling best-hit of the HG002-HPRCy1 contigs with the best-hit supported by HG002-Verkko's contigs. We considered only best-hits with an estimated identity of at least 90% that cover regions labeled as reliable. We reported the number of different targets for each reference position. On the bottom, the untangling of HG002's contigs from HG002-Verkko assembly as those seen in HG002-HPRCy1 assemblies, for chromosome 14 versus T2T-CHM13. Transparency shows the estimated identity of the mappings. We display all mappings above 90% estimated pairwise identity. Checkerboard patterns observed in several regions of the SAACs correspond to contexts that may permit ongoing recombination.

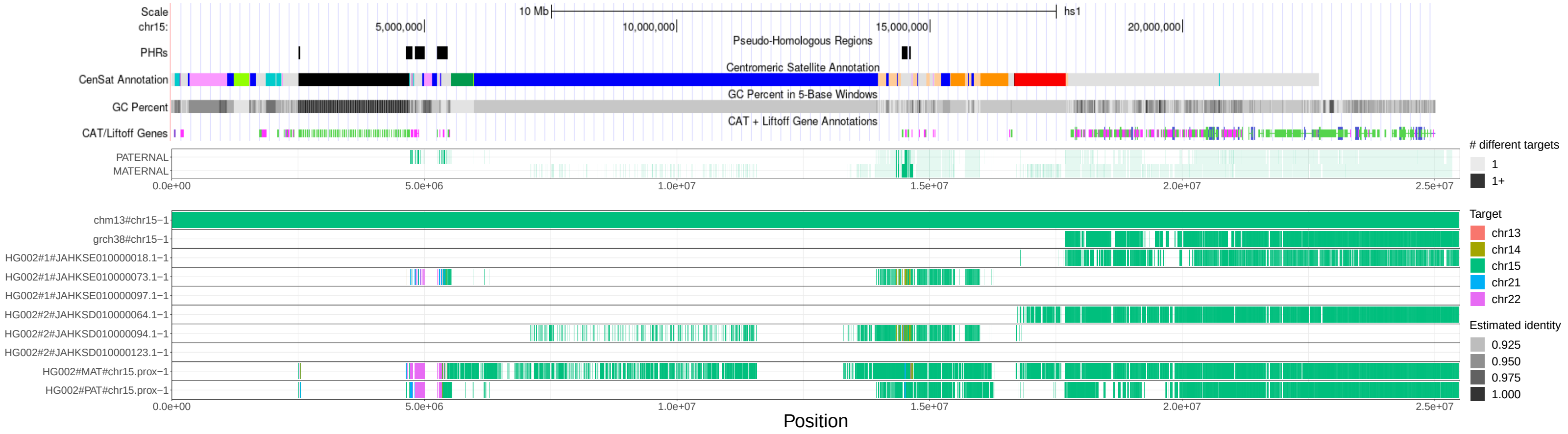

Supplementary Figure 11: For each base position of T2T-CHM13 chromosome 15, we compared the untangling best-hit of the HG002-HPRCy1 contigs with the best-hit supported by HG002-Verkko's contigs. We considered only best-hits with an estimated identity of at least 90% that cover regions labeled as reliable. We reported the number of different targets for each reference position. On the bottom, the untangling of HG002's contigs from HG002-Verkko assembly as those seen in HG002-HPRCy1 assemblies, for chromosome 15 versus T2T-CHM13. Transparency shows the estimated identity of the mappings. We display all mappings above 90% estimated pairwise identity. Checkerboard patterns observed in several regions of the SAACs correspond to contexts that may permit ongoing recombination.

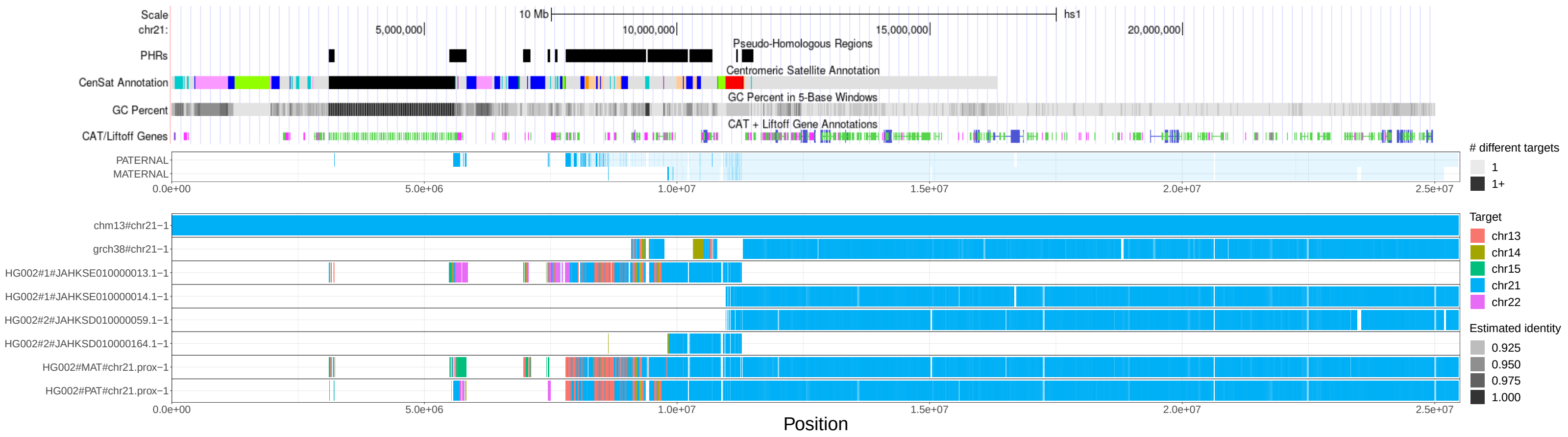

Supplementary Figure 12: For each base position of T2T-CHM13 chromosome 21, we compared the untangling best-hit of the HG002-HPRCy1 contigs with the best-hit supported by HG002-Verkko's contigs. We considered only best-hits with an estimated identity of at least 90% that cover regions labeled as reliable. We reported the number of different targets for each reference position. On the bottom, the untangling of HG002's contigs from HG002-Verkko assembly as those seen in HG002-HPRCy1 assemblies, for chromosome 21 versus T2T-CHM13. Transparency shows the estimated identity of the mappings. We display all mappings above 90% estimated pairwise identity. Checkerboard patterns observed in several regions of the SAACs correspond to contexts that may permit ongoing recombination.

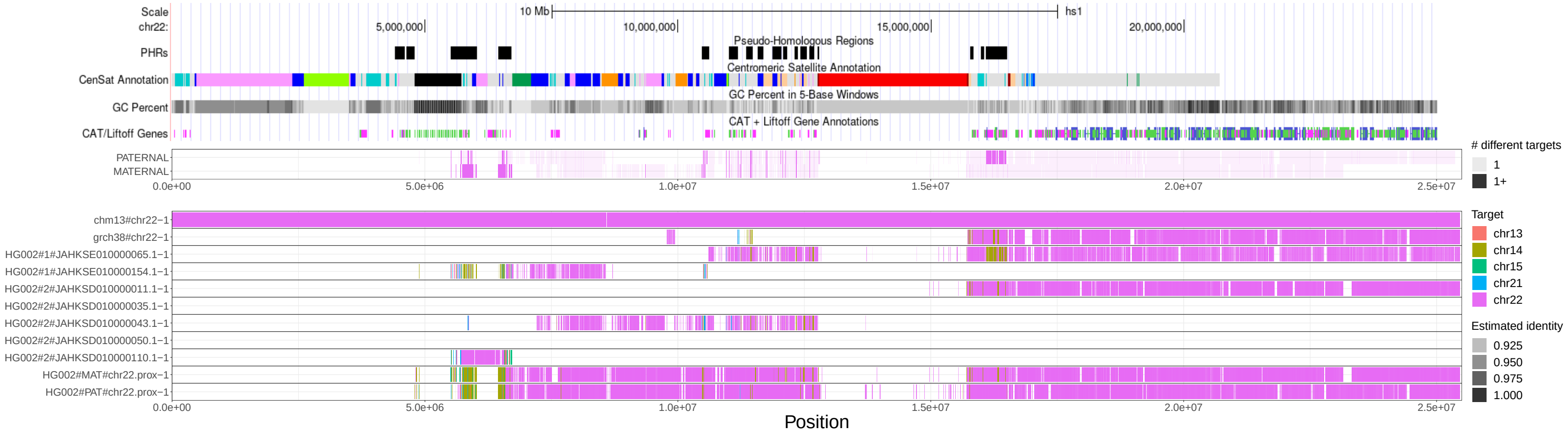

Supplementary Figure 13: For each base position of T2T-CHM13 chromosome 22, we compared the untangling best-hit of the HG002-HPRCy1 contigs with the best-hit supported by HG002-Verkko's contigs. We considered only best-hits with an estimated identity of at least 90% that cover regions labeled as reliable. We reported the number of different targets for each reference position. On the bottom, the untangling of HG002's contigs from HG002-Verkko assembly as those seen in HG002-HPRCy1 assemblies, for chromosome 22 versus T2T-CHM13. Transparency shows the estimated identity of the mappings. We display all mappings above 90% estimated pairwise identity. Checkerboard patterns observed in several regions of the SAACs correspond to contexts that may permit ongoing recombination.

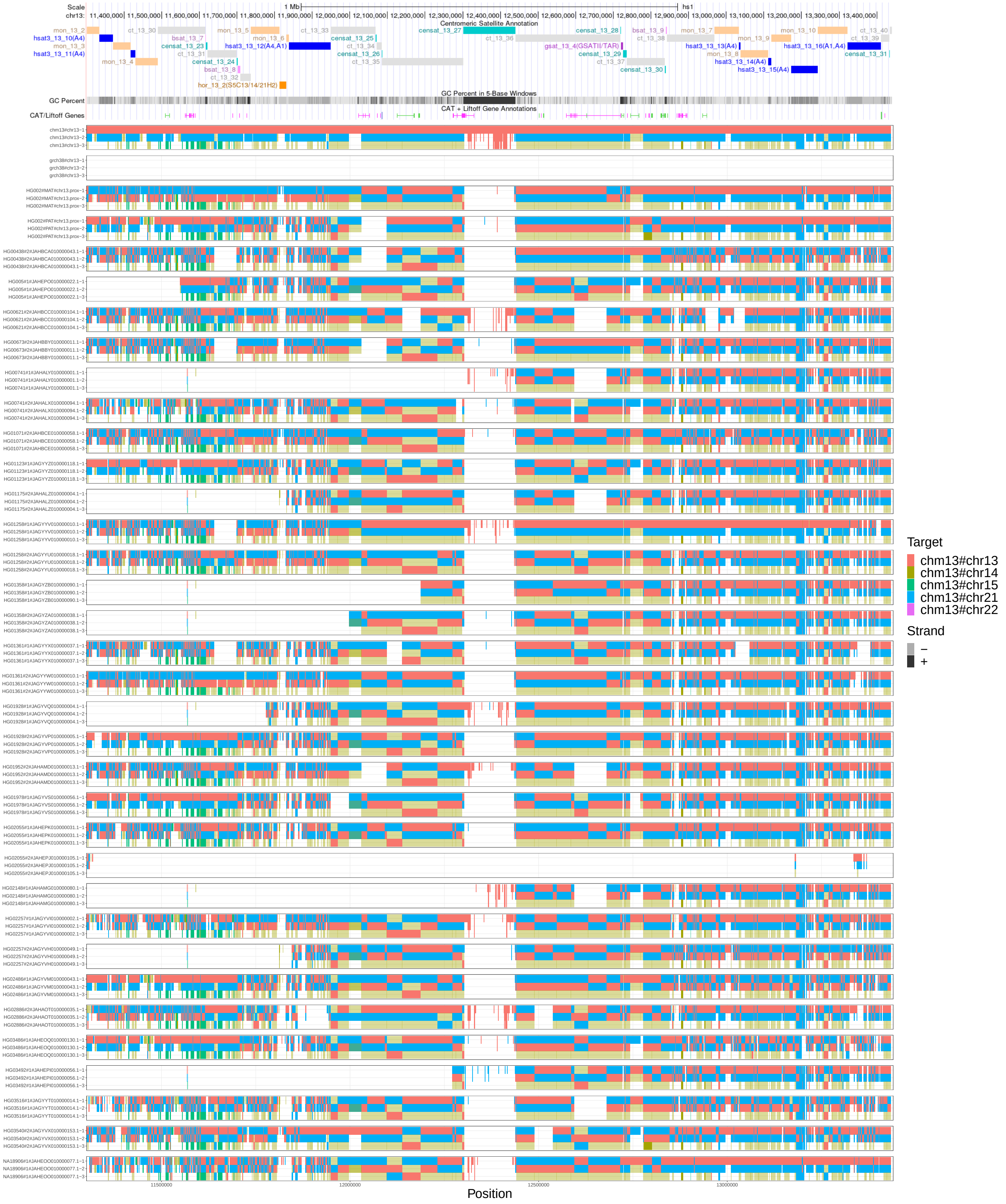

Supplementary Figure 14: Multiple untangling of T2T-CHM13, GRCh38, HG002-Verkko haplotypes, and HPRCy1-acro contigs versus T2T-CHM13. Chromosome 13 results are represented, in the chr13:11,301,367-13,440,010 region (censat_13_27 coordinates +- 1Mbp). Transparency shows the different orientations of the mappings. We display all mappings above 90% estimated pairwise identity. To analyze simultaneous hits to all acrocentrics, each grouping shows the first 3 best alternative mappings. The figure shows that chromosome 13's contigs map in forward orientation on T2T-CHM13 chromosome 13 and 21 (orange and cyan rectangles), while their mappings are inverted on chromosomes 14 (transparent gold rectangles).

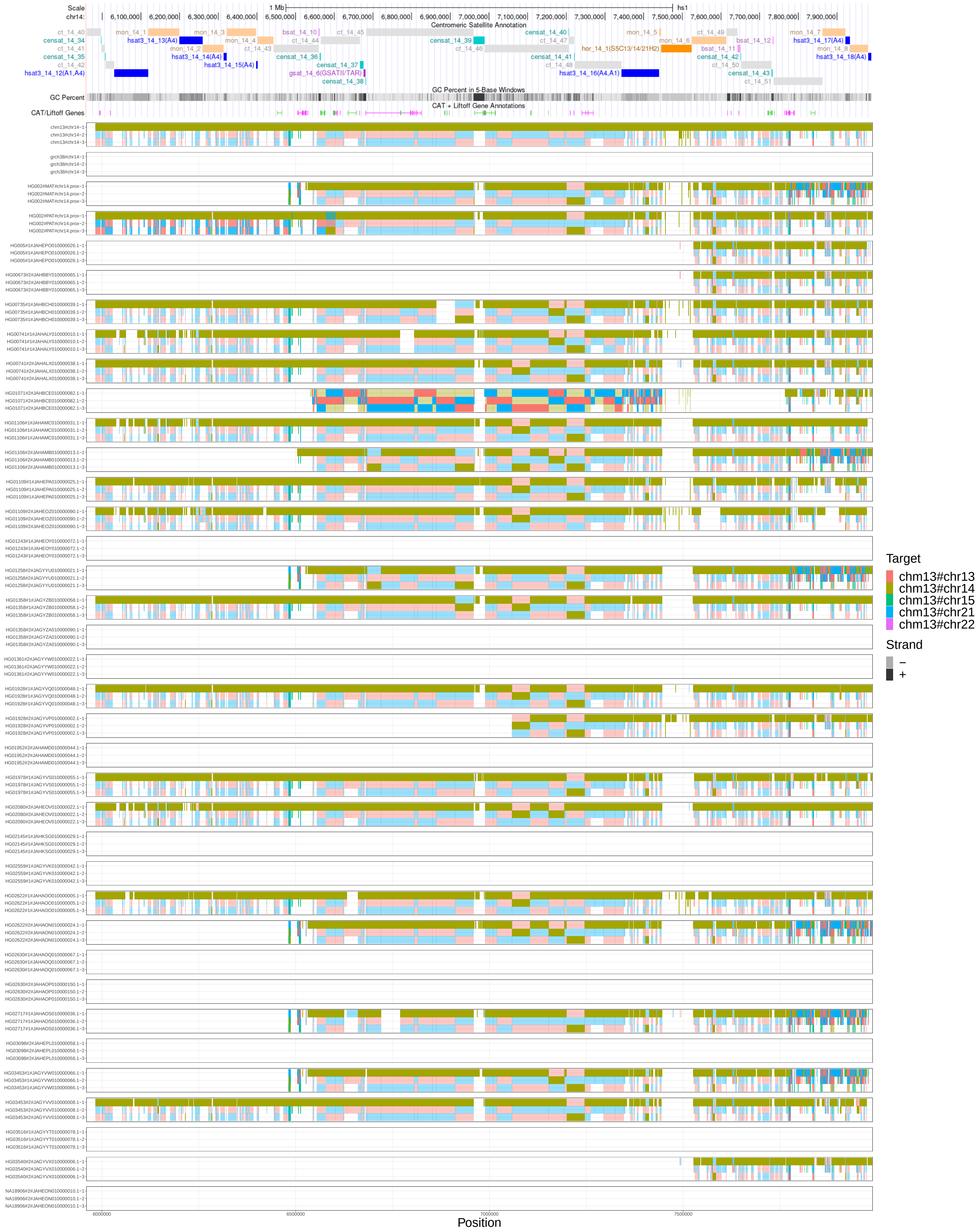

Supplementary Figure 15: Multiple untangling of T2T-CHM13, GRCh38, HG002-Verkko haplotypes, and HPRCy1-acro contigs versus T2T-CHM13. Chromosome 14 results are represented, in the chr14:5,960,008-7,988,409 region (censat_14_39 coordinates +- 1Mbp). Transparency shows the different orientations of the mappings. We display all mappings above 90% estimated pairwise identity. To analyze simultaneous hits to all acrocentrics, each grouping shows the first 3 best alternative mappings. The figure shows that chromosome 14's contigs map in forward orientation on T2T-CHM13 chromosome 14 (gold rectangles), while their mappings are inverted on chromosomes 13 and 21 (transparent orange and cyan rectangles), with the sole exception of HG01071#2#JAHBCE010000082.1, where the trend is reversed.

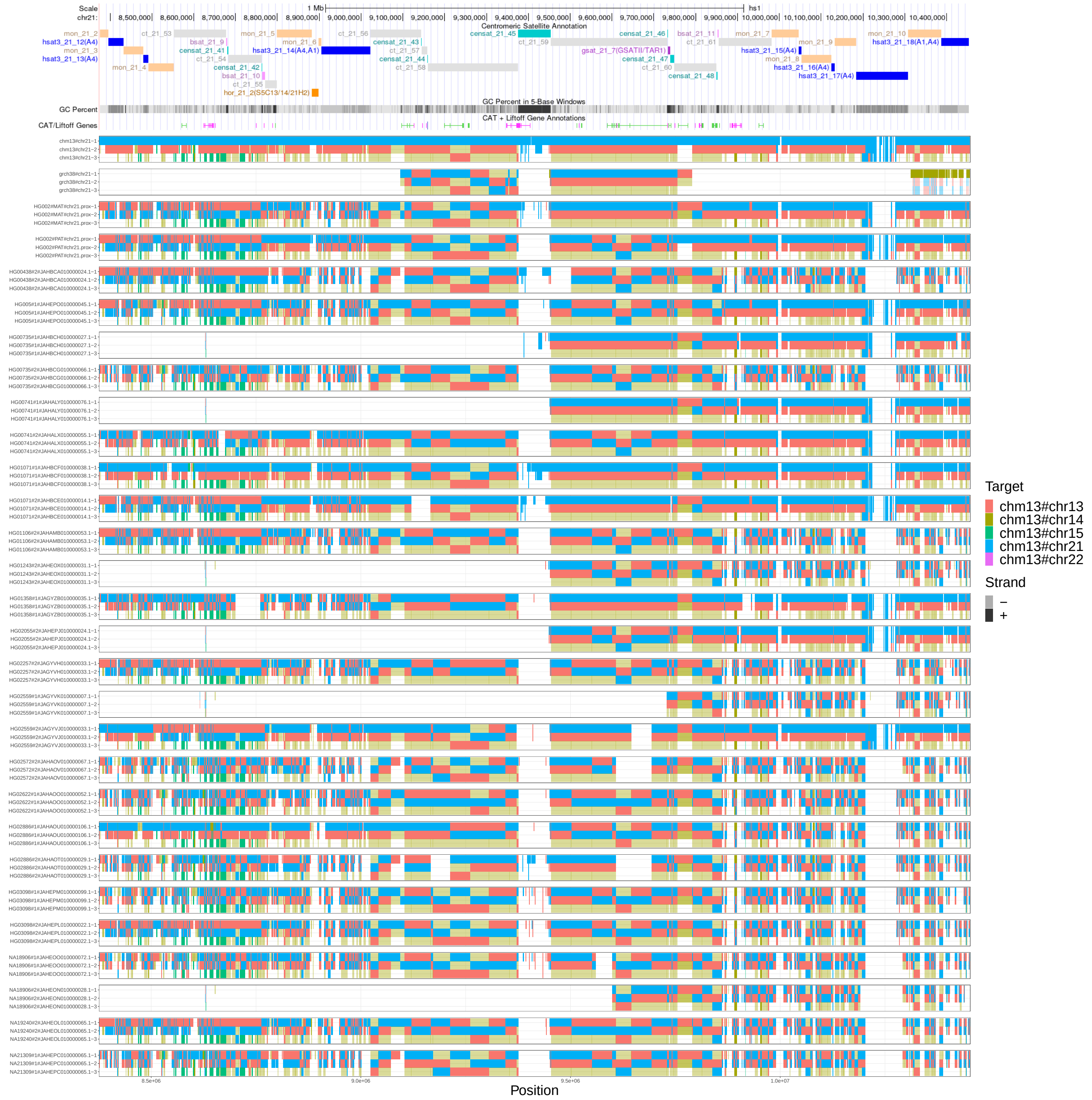

Supplementary Figure 16: Multiple untangling of T2T-CHM13, GRCh38, HG002-Verkko haplotypes, and HPRCy1-acro contigs versus T2T-CHM13. Chromosome 21 results are represented, in the chr21:8,375,567-10,453,313 region (censat_21_45 coordinates +- 1Mbp). Transparency shows the different orientations of the mappings. We display all mappings above 90% estimated pairwise identity. To analyze simultaneous hits to all acrocentrics, each grouping shows the first 3 best alternative mappings. The figure shows that chromosome 21's contigs map in forward orientation on T2T-CHM13 chromosome 21 and 13 (cyan and orange rectangles), while their mappings are inverted on chromosomes 14 (transparent gold rectangles).

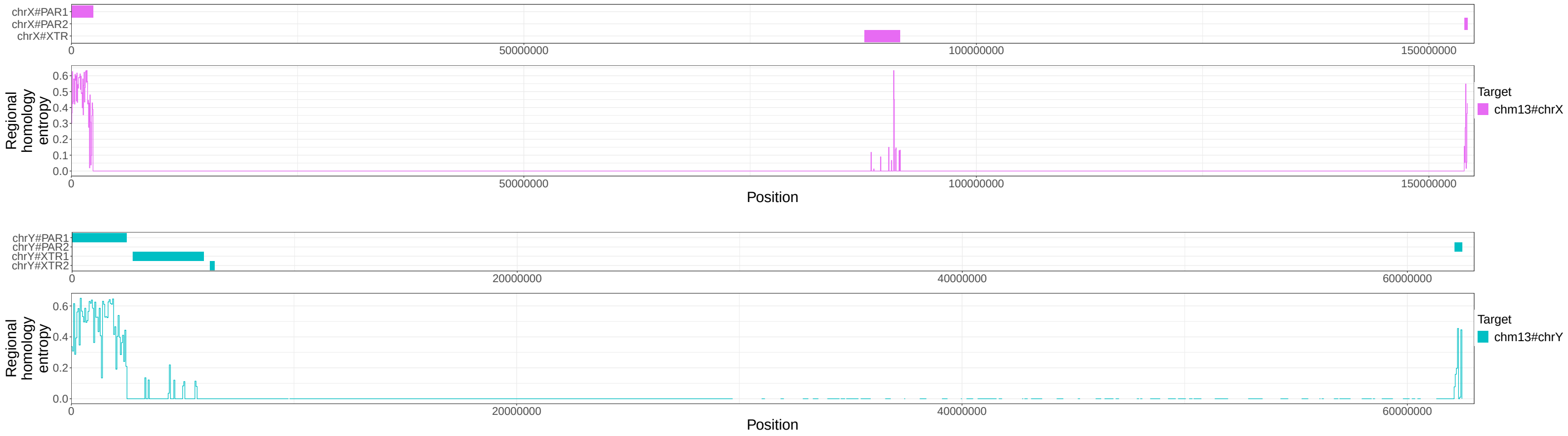

Supplementary Figure 17: For HPRCy1 contigs from chromosome X and Y, we show the tracks for the pseudoautosomal regions (PARs) and the X-transposed region (XTRs) with respect to T2T-CHM13 (on the top) as well as the untangle entropy metric (Regional homology metric, on the bottom) computed over the contigs' T2T-CHM13-relative untanglings.

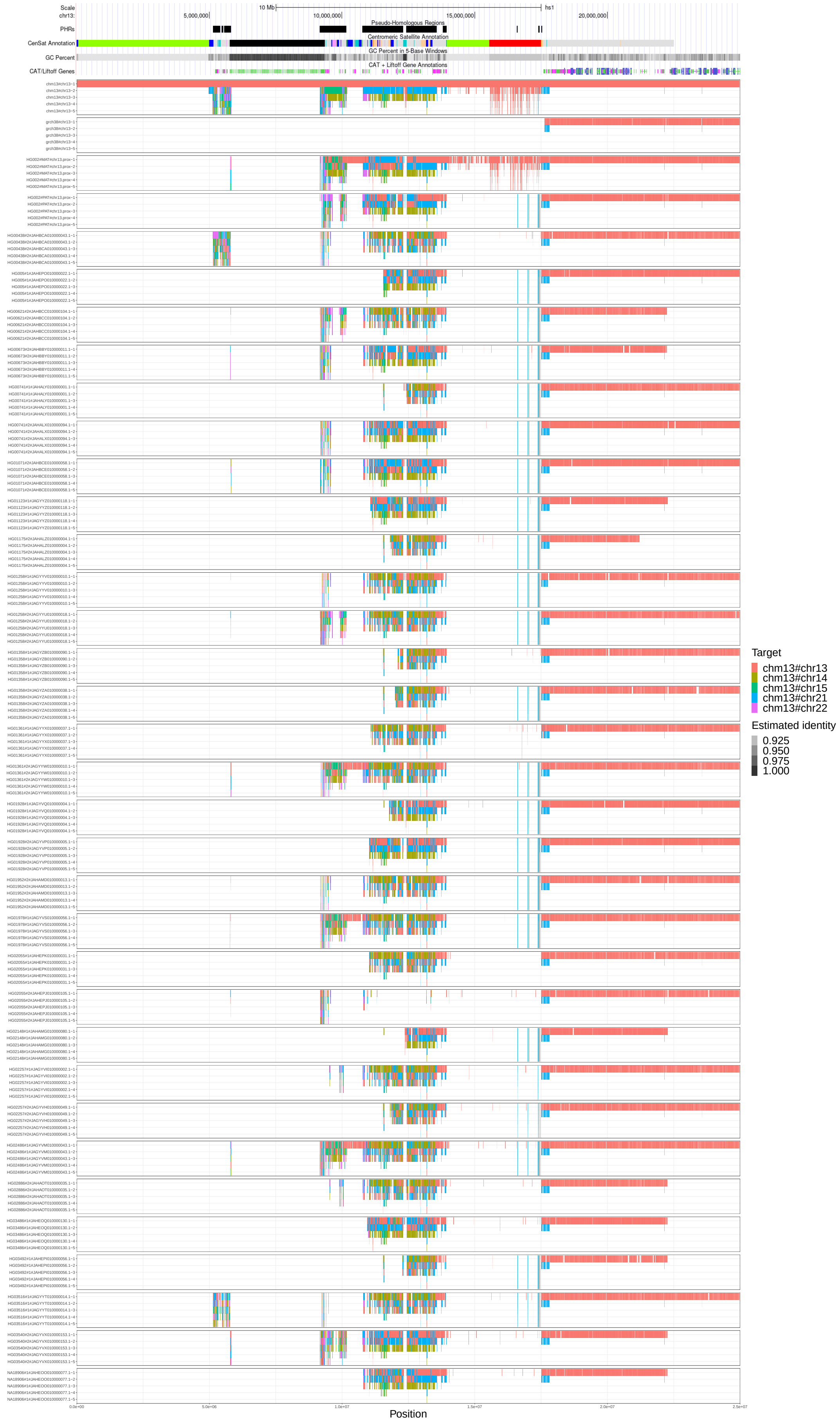

Supplementary Figure 18: Multiple untangling of HPRCy1-acro's sequences belonging to chromosome 13 versus T2T-CHM13. Transparency shows the estimated identity of the mappings. We display all mappings above 90% estimated pairwise identity that cover regions labeled as reliable. To allow the display of simultaneous hits to all acrocentrics, each grouping shows the first 5 best alternative mappings. Checkerboard patterns observed in several regions of the SAACs correspond to contexts that may permit ongoing recombination.

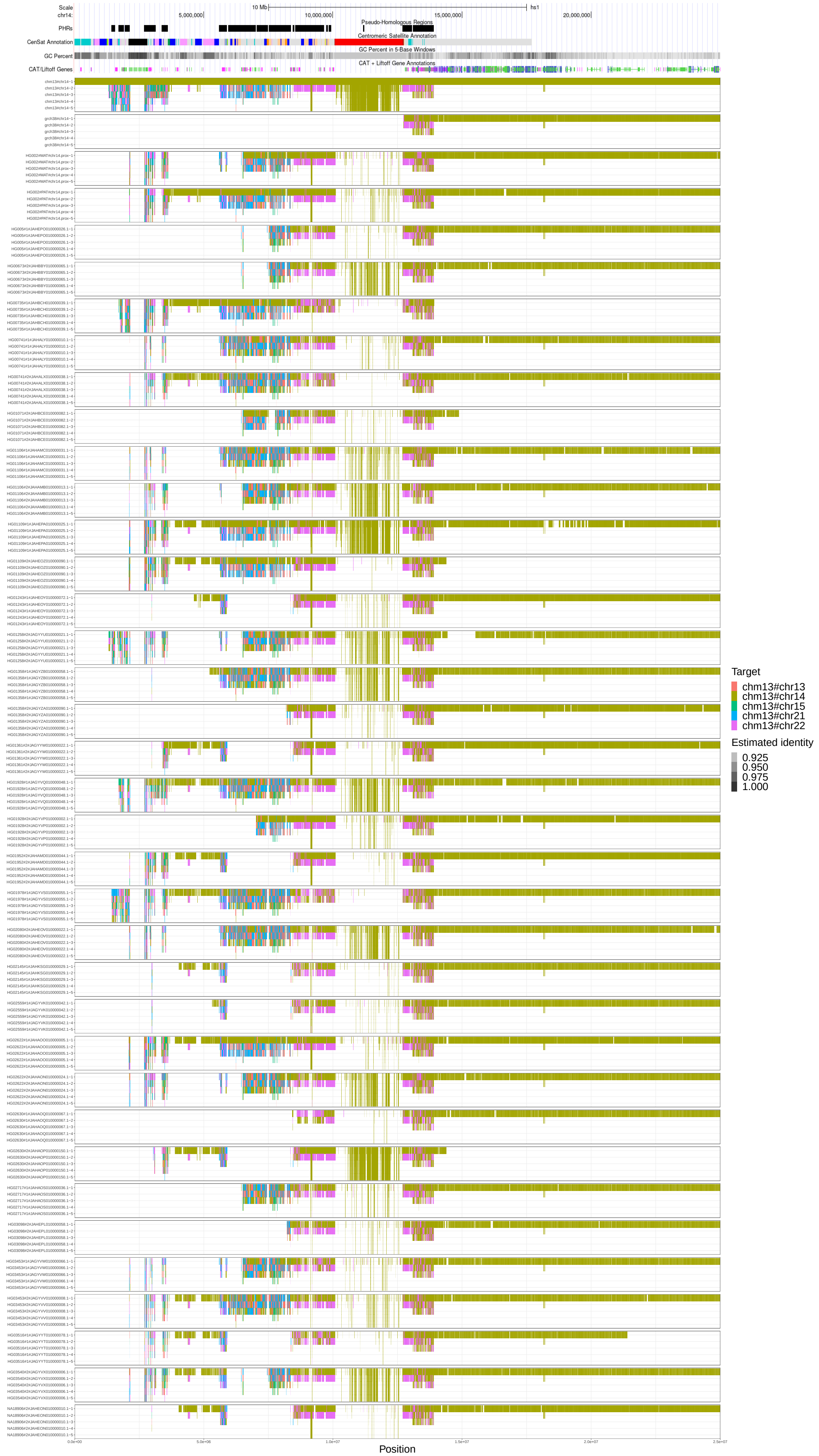

Supplementary Figure 19: Multiple untangling of HPRCy1-acro's sequences belonging to chromosome 14 versus T2T-CHM13. Transparency shows the estimated identity of the mappings. We display all mappings above 90% estimated pairwise identity that cover regions labeled as reliable. To allow the display of simultaneous hits to all acrocentrics, each grouping shows the first 5 best alternative mappings. Checkerboard patterns observed in several regions of the SAACs correspond to contexts that may permit ongoing recombination.

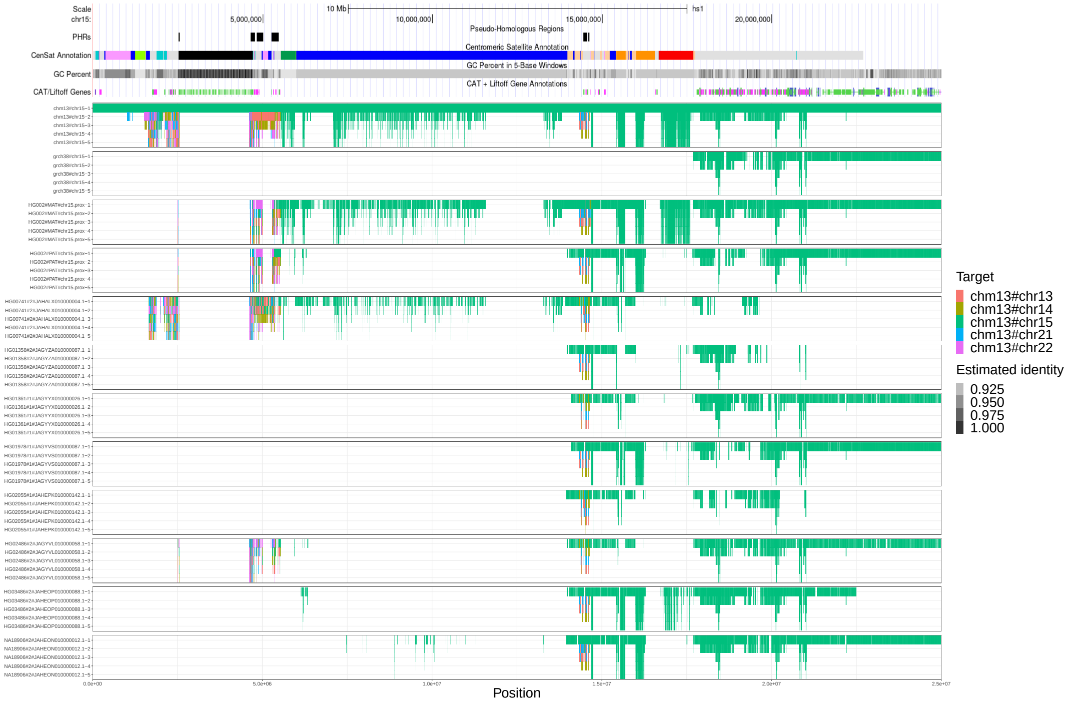

Supplementary Figure 20: Multiple untangling of HPRCy1-acro's sequences belonging to chromosome 15 versus T2T-CHM13. Transparency shows the estimated identity of the mappings. We display all mappings above 90% estimated pairwise identity that cover regions labeled as reliable. To allow the display of simultaneous hits to all acrocentrics, each grouping shows the first 5 best alternative mappings. Checkerboard patterns observed in several regions of the SAACs correspond to contexts that may permit ongoing recombination.

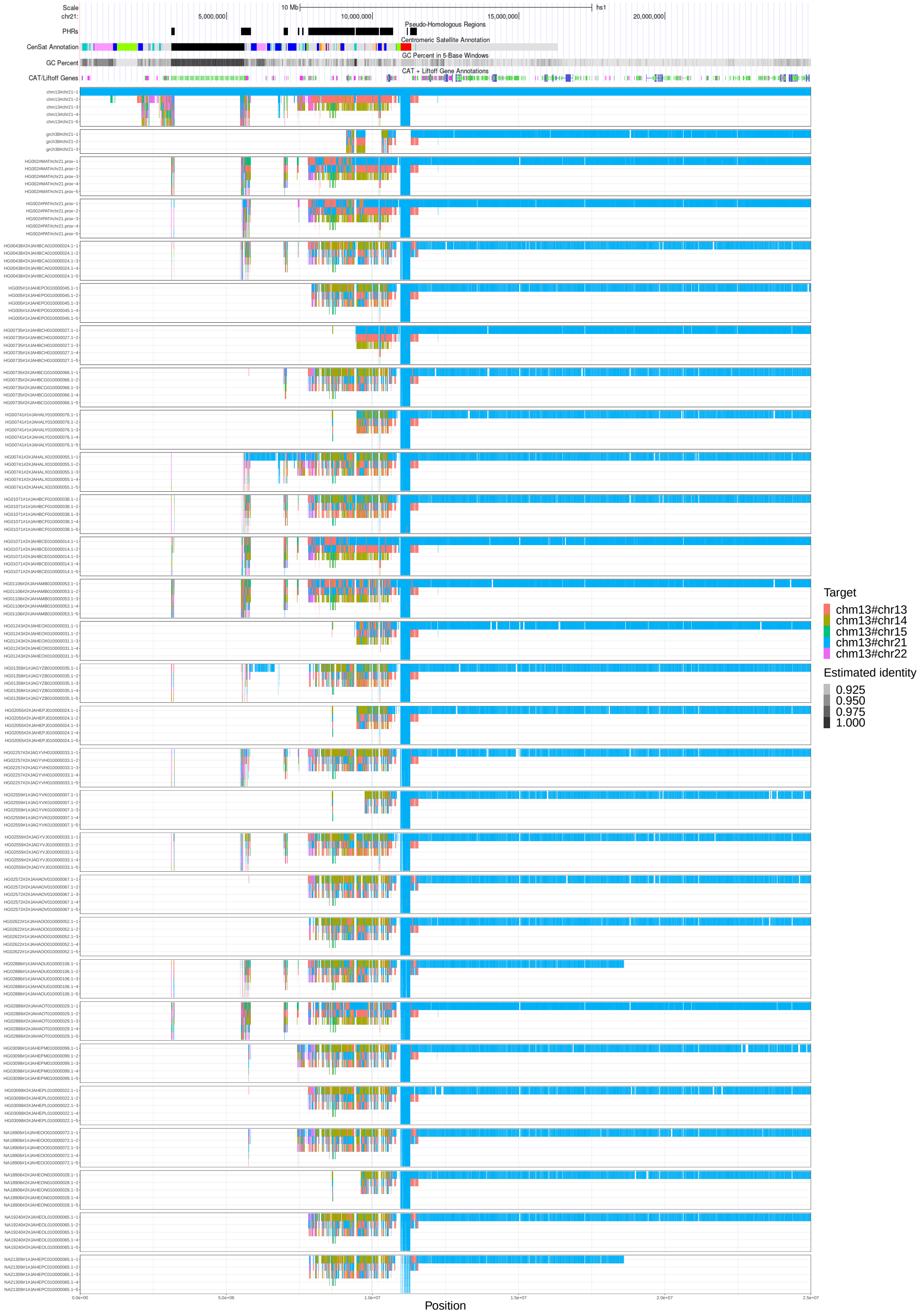

Supplementary Figure 21: Multiple untangling of HPRCy1-acro's sequences belonging to chromosome 21 versus T2T-CHM13. Transparency shows the estimated identity of the mappings. We display all mappings above 90% estimated pairwise identity that cover regions labeled as reliable. To allow the display of simultaneous hits to all acrocentrics, each grouping shows the first 5 best alternative mappings. Checkerboard patterns observed in several regions of the SAACs correspond to contexts that may permit ongoing recombination.

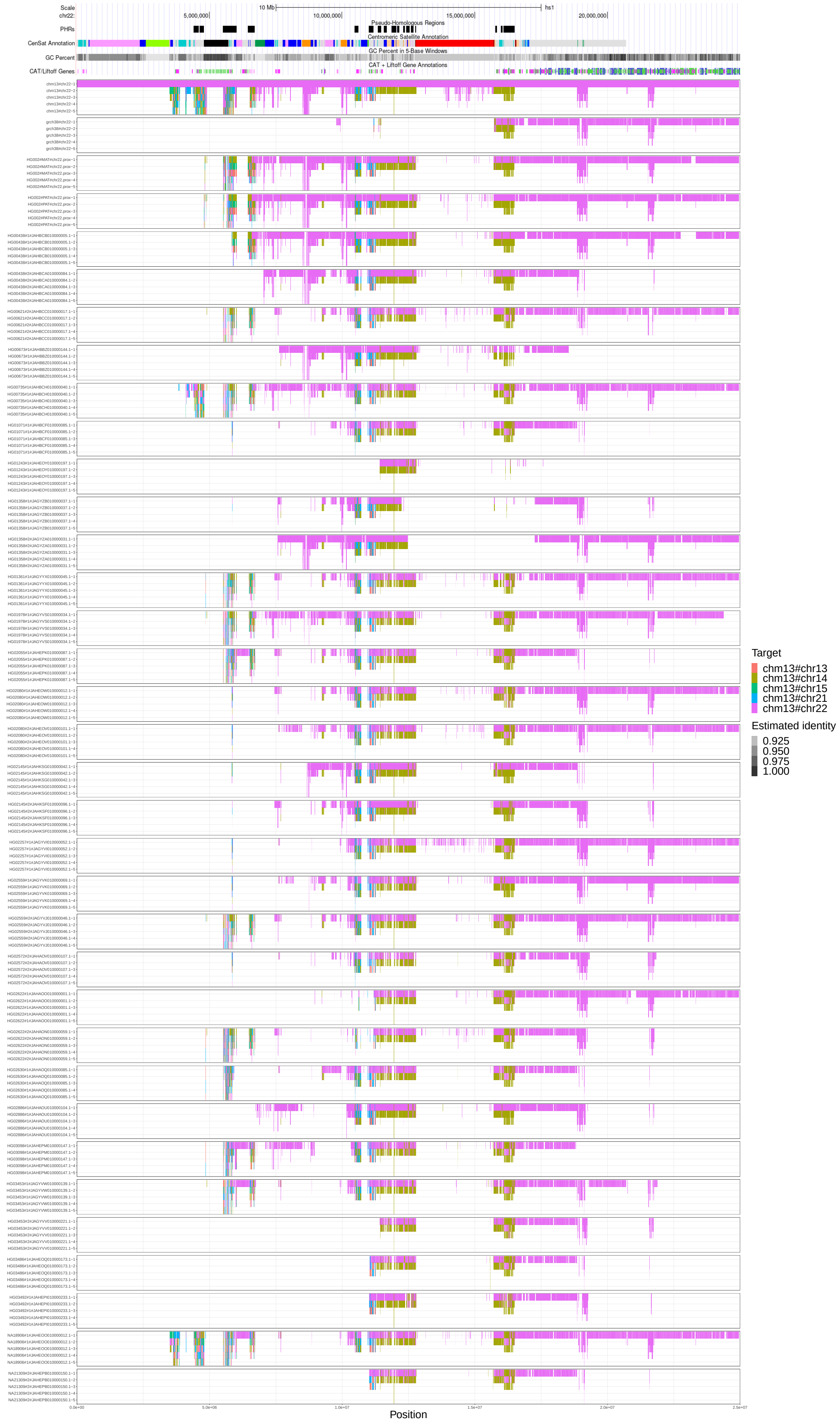

Supplementary Figure 22: Multiple untangling of HPRCy1-acro's sequences belonging to chromosome 22 versus T2T-CHM13. Transparency shows the estimated identity of the mappings. We display all mappings above 90% estimated pairwise identity that cover regions labeled as reliable. To allow the display of simultaneous hits to all acrocentrics, each grouping shows the first 5 best alternative mappings. Checkerboard patterns observed in several regions of the SAACs correspond to contexts that may permit ongoing recombination.

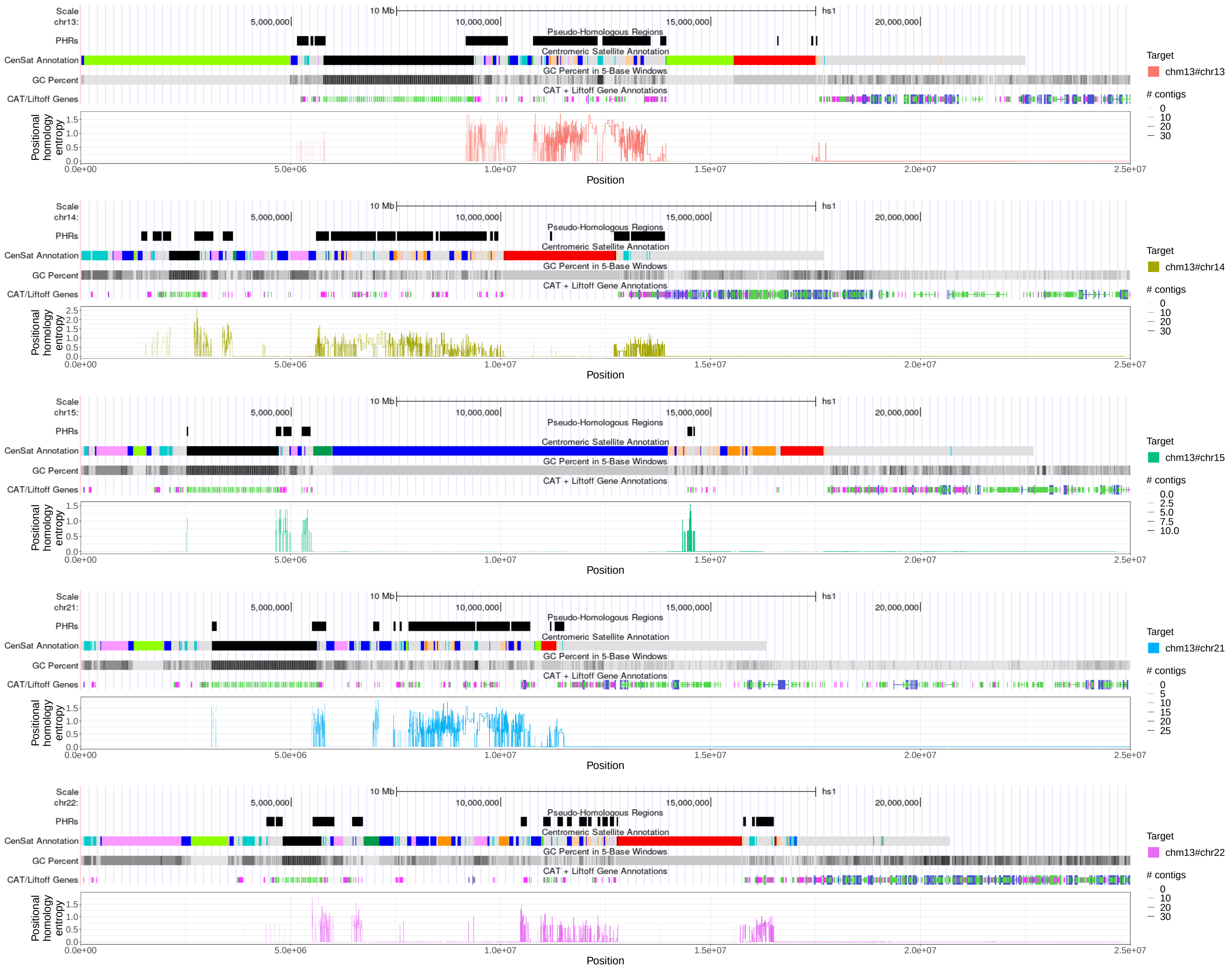

Supplementary Figure 23: Multi-hit untangling diversity entropy for HPRCy1-acro's sequences belonging to chromosome 13, 14, 15, 21, 22 versus T2T-CHM13. We considered all mappings above 90% estimated pairwise identity that cover regions labeled as reliable.

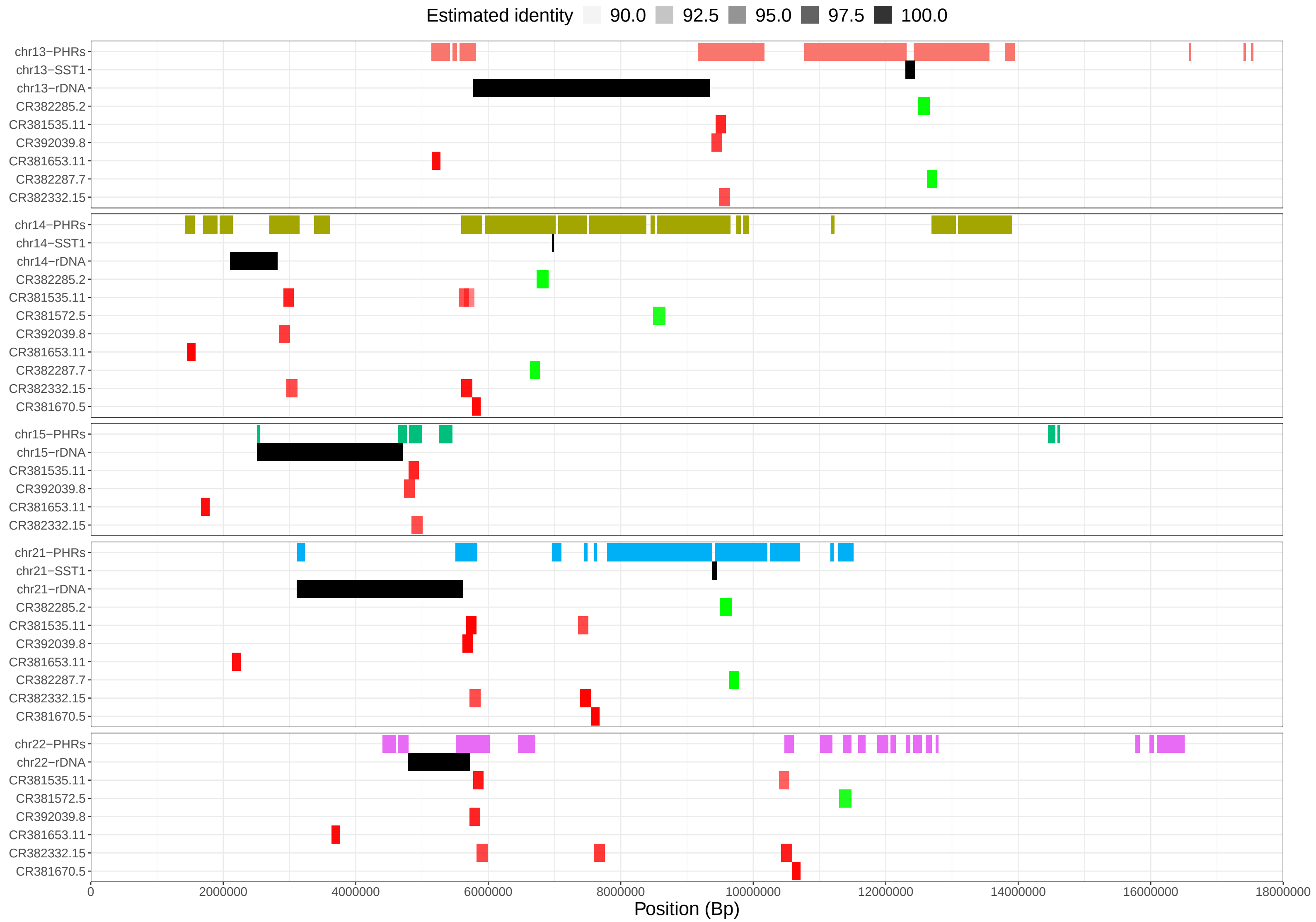

Supplementary Figure 24: For each T2T-CHM13 acrocentric chromosome, we show the tracks for the pseudo-homologous regions (PHRs), the SST1 array, the rDNA arrays, and for the regions where the bacterial artificial chromosome (BAC) clones from (Jarmuz-Szymczak et al. 2014) map on those chromosomes. Most of the mappings cover the PHRs. The mappings with higher estimated identity (¿ 99%) are on chr21 and chr14. We colored BAC clones' mappings according to (Jarmuz-Szymczak et al. 2014).

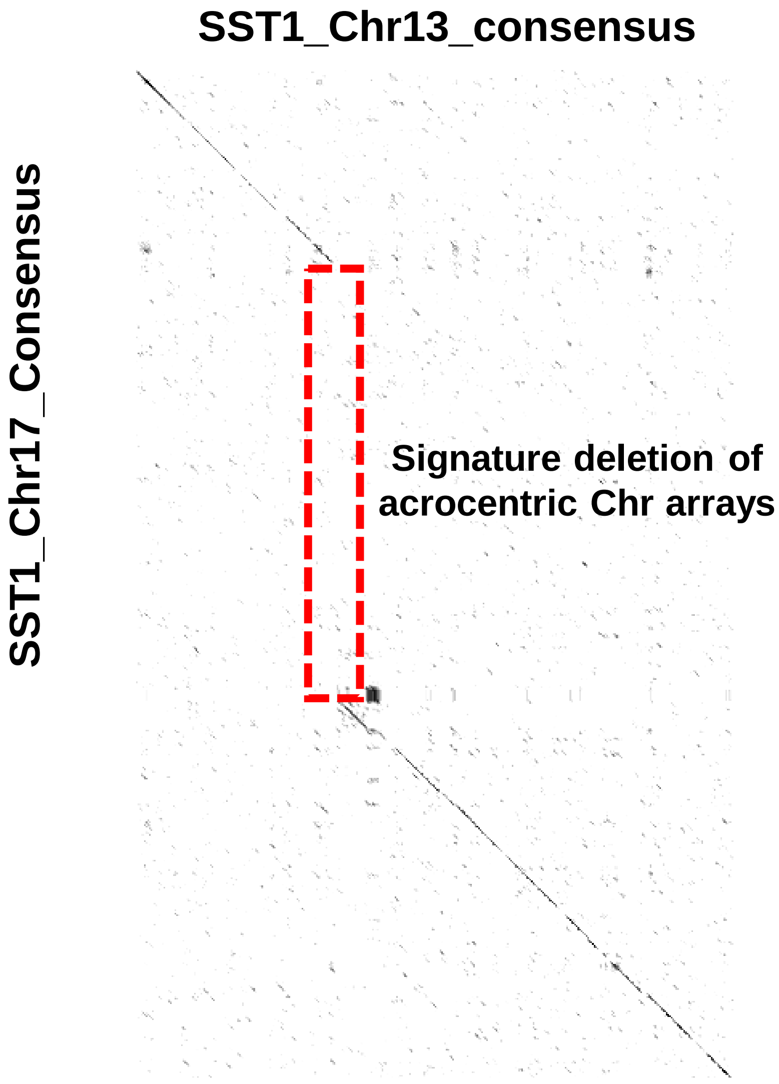

Supplementary Figure 25: Comparison of chr13 and chr17 SST1 consensus sequences by alignment dotplot indicate the unique deletion shared exclusively by the acrocentric chromosomes (dotted red rectangle).

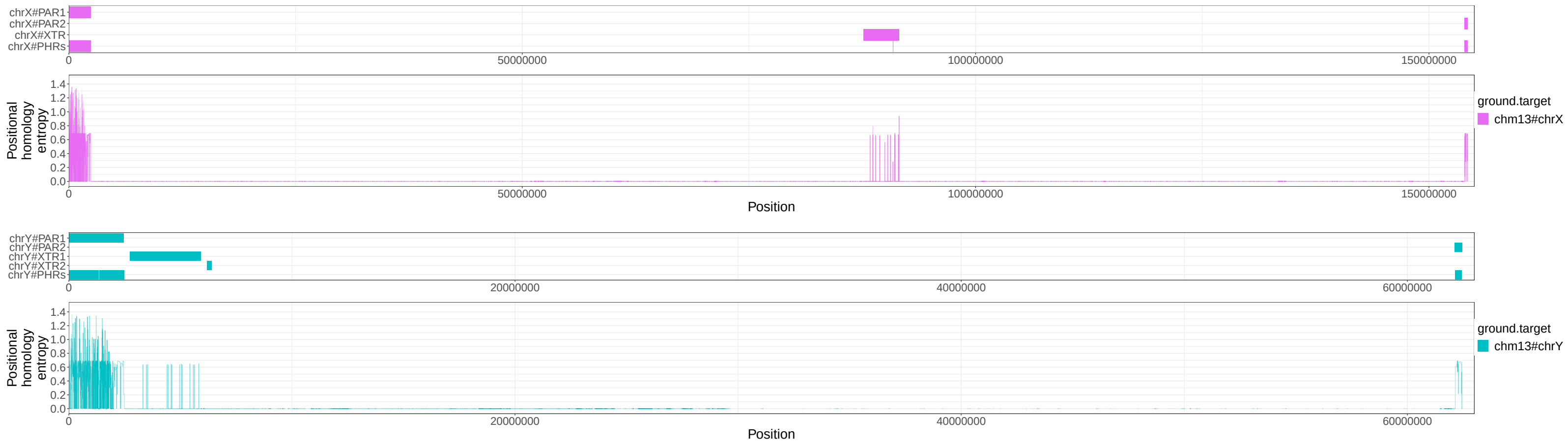

Supplementary Figure 26: For HPRCy1 contigs from chromosome X and Y, we show the tracks for the pseudoautosomal regions (PARs) and the X-transposed region (XTRs) with respect to T2T-CHM13 as well as the multi untangle entropy metric (Positional homology entropy, on the bottom) computed over the contigs' T2T-CHM13-relative untanglings.

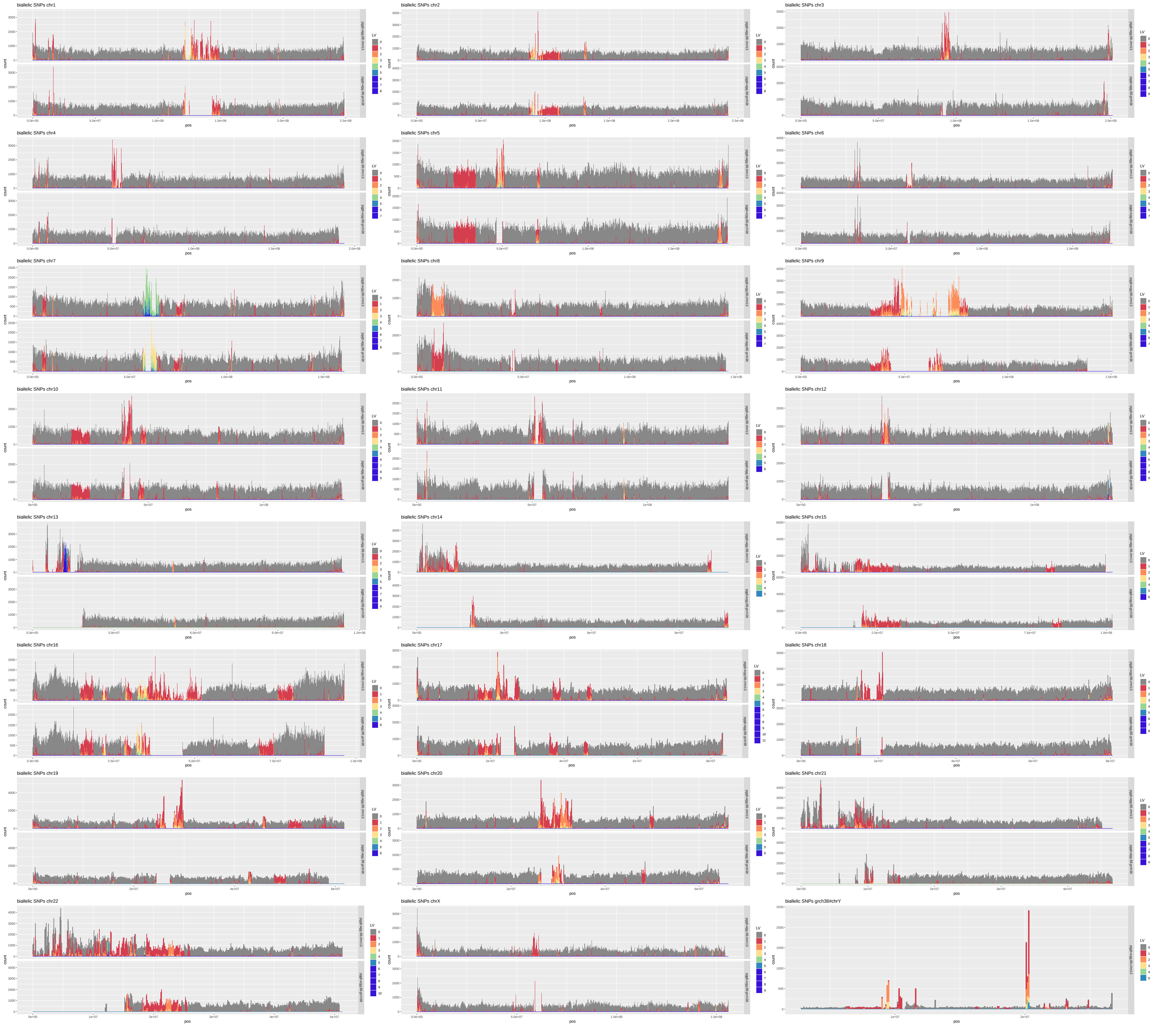

Supplementary Figure 27: Biallelic SNP density in the full HPRCy1 draft pangenome. Colors indicate the count of SNPs in each bin at given variant nesting levels (Paten et al. 2018), with gray showing the base level and a red to violet spectrum for increasing level.

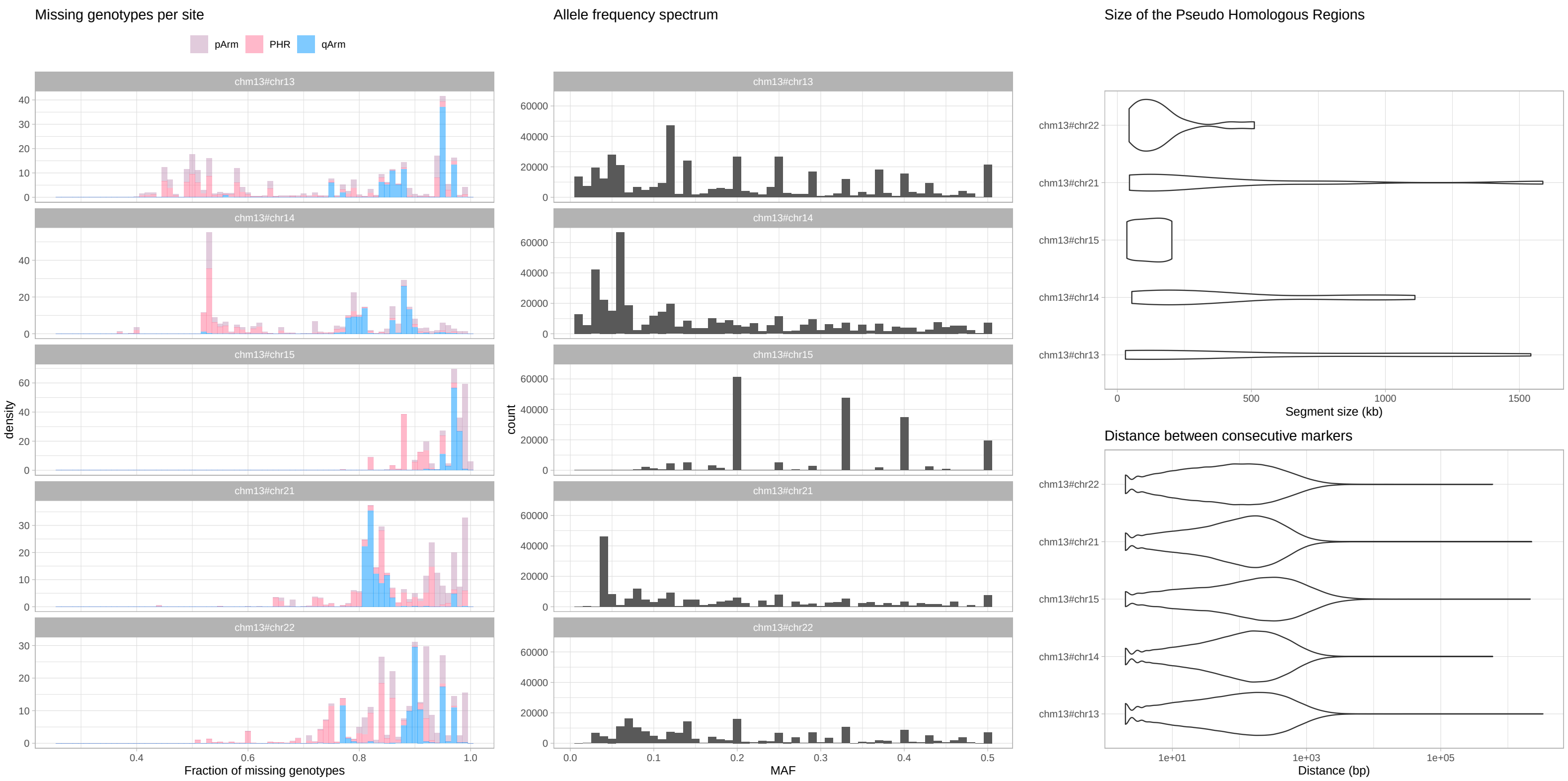

Supplementary Figure 28: Left) Histograms showing missing genotypes per site for variants called with respect to the T2T-CHM13 acrocentrics. Middle) Histograms showing the allele frequency spectrum for variants called with respect to the T2T-CHM13 acrocentrics. MAF = Minor Allele Frequency. Right-Top) Segment size distributions of the pseudo-homologous regions with respect to the T2T-CHM13 acrocentrics. Right-Bottom) Distributions of the distance between consecutive markers with respect to the T2T-CHM13 acrocentrics.

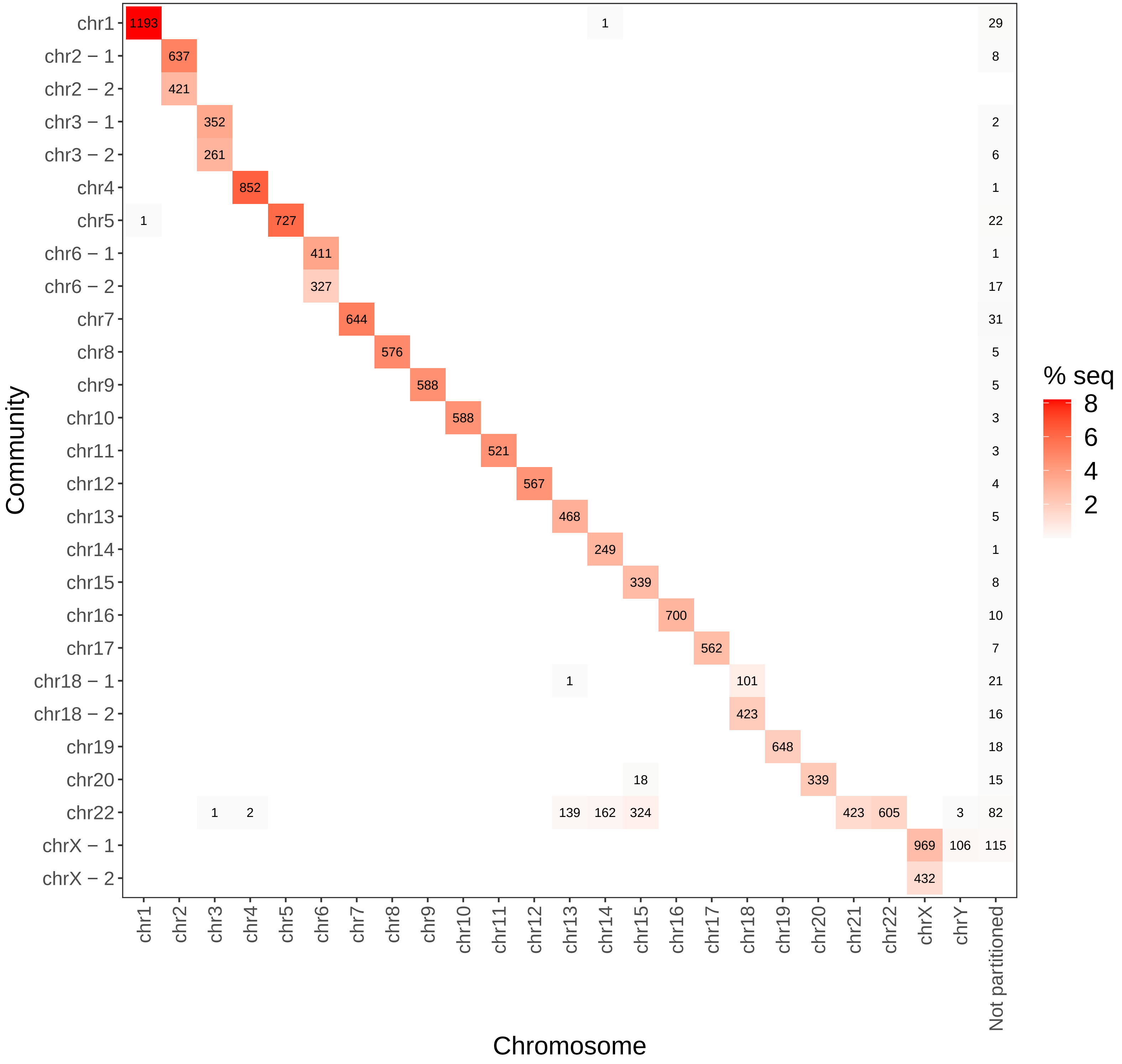

Supplementary Figure 29: Results of community assignment on the mapping graph when using chains of 10 kbp seeds of 95% average nucleotide identity. On the x-axis the chromosome to which contigs belong based on competitive mapping to T2T-CHM13 and GRCh38, while the y-axis indicates the community, which is named by the chromosome partition that contributes the largest number of contigs to it.
