# Supplementary material for: Recombination between heterologous human acrocentric chromosomes
Source: Nature. 2023 May 10;617(7960):335–43. doi: 10.1038/s41586-023-05976-y (PMC10172130; doi:10.1038/s41586-023-05976-y)
Supplement: Supplementary file 5 — This file contains Supplementary Files 1–8. [file 41586_2023_5976_MOESM5_ESM.docx]

**Supplementary File Legends for**

**Recombination between heterologous human acrocentric chromosomes**

All supplementary files are available on Zenodo at <https://doi.org/10.5281/zenodo.7692554>.

**Supplementary File 1.** Results of community assignment on the mapping graph. The 'community.of' column reports the community names assigned by the chromosomal partition that contributes the most contigs. Columns 'chr1' to 'chrY' report the number of contigs assigned to each chromosome based on the competitive mapping to the chromosomes of T2T-CHM13 and GRCh38. The 'non.partitioned' column reports the number of contigs not mappable to T2T-CHM13 and GRCh38.

**Supplementary File 2.** Acrocentric pangenome variation graph in GFAv1 file format (<https://github.com/GFA-spec/GFA-spec>).

**Supplementary File 3.** Graph layout in 2 dimensions. The first column is an incremental number, the second and third columns are the X and Y coordinates and the fourth column identifies the graph connected component.

**Supplementary File 4.** For each HPRCy1-acro contig (‘contig’ column), we report the amount of projected alignments (‘untangled.size’ column), the amount of reliable projected alignments (‘reliable.untangled.size’ column), and the fraction of unreliable alignments that we ignored in our analysis (‘fraction.removed’ column). Regions are labeled as reliable or unreliable by FLAGGER.

**Supplementary File 5.** Pseudo-homologous regions identified in the acrocentric chromosomes of T2T-CHM13. The first three columns represent the T2T-CHM13 chromosome, the starting position, and the ending position of each pseudo-homologous region. Column 4 is the average Shannon Diversity Index. Column 5 is the average number of contigs.

**Supplementary File 6.** Pseudo-homologous regions identified in the sex chromosomes of T2T-CHM13. The first three columns represent the T2T-CHM13 chromosome, the starting position, and the ending position of each pseudo-homologous region. Column 4 is the average Shannon Diversity Index. Column 5 is the average number of contigs.

**Supplementary File 7.** PRDM9 binding motif hits in T2T-CHM13. Column 1 and 2 identify the motif. Column 3, 4, 5 indicate the sequence on which the motif has been found, with the start and end coordinates. Column 6 reports the strand of the hit on the sequence. Column 7 is the score of the motif hit. Column 8 and 9 report, respectively, the p-value and the adjusted p-value. Column 10 is the matched sequence.

**Supplementary File 8.** For each T2T-CHM13 chromosome (‘ground.target’ column), we report the number of bases where HG002-HiFi and HG002-Verkko assemblies are concordant and discordant (‘num.bp.ok’ and ‘num.bp.not.ok’ columns), and the percentage of concordant bases (‘percentage.bp.ok’ column). Concordant bases are those where the untangling best-hits from both assemblies is on the same T2T-CHM13 chromosome. Values are reported for both maternal and paternal haplotypes (‘haplotype’ column) and stratified by chromosomal arm or whole region (‘region’ column).
